# A case of eosinophilic esophagitis, with sensitization to BSA (meat allergy) and rhinoconjunctivitis due to dog epithelium

**DOI:** 10.1186/2045-7022-3-S3-P166

**Published:** 2013-07-25

**Authors:** L Vazquez Fuertes, M Perez Bustamante, A Bueso Fernandez, A Gonzalez Pino, F Pineda De La Losa

**Affiliations:** 1 Allergy, Torrejon Hospital, Madrid, Spain; 2 Gastroenterology, Torrejon Hospital, Madrid, Spain; 3 Laboratory Department, Diater Laboratory, Madrid, Spain

## Background

Patients with eosinophilic esophagitis (EEo) have a prior history of atopy. Skin prick test(SPT) is able to identify the allergens involved, allowing a correct dietary approach, in order to achieve the remission of sympthoms and discontinue swallowed costicostheroids. Allergy to bovine meat and especially to Bovine serum albumin (BSA) is exceptional in adult life, but can be a cause of EEo.

## Methods

We present one 26-year-old patient with EEo diagnosed six months before, who presented characteristic sympthoms after eating mamal´s meat (well cooked beef and pork), and legumes (chickpea), as food impactation. She had history of rhinitis, related to sensitization to dog epithelium. The patient underwent SPT, specific IgE detection and SDS-PAGE immunoblotting studies.

## Results

The SPT with food allergens showed negative responses to pork, cow, rabbit, lamb, chicken meat, milk, egg, legumes (including chickpea), nuts, fruits and vegetables, fish, seafood, cereals. It showed positive result to BSA. The SPT with aeroallergens showed negative responses to all we studied, but dog epithelium that was positive. The determination of specific IgE were negative to beef and pig meat,milk,egg, legumes, nuts, cereals. The result was positive to dog epithelium (17.1 ku/l CAP), dog BSA (8.03 ku/l), cow BSA (0.51 ku/l). Total IgE 214UI/l.IgE-Immunoblotting indicates recognition bands of 60-70 kDa components (molecular weight compatible with BSA) in dog epithelium extract, and less intensity response in meat extracts. Two allergens with a molecular weight close to 15-20 kDa were recognized. We found reactivity to 40-45 KDa allergens in meat extract also.

## Conclusion

We report a case of EEo, who had sensitization to BSA and dog epithelium. The patient suffered previous rhinoconjunctivitis related to her dog, but we could not demonstrate cross-reactivity. It is suggested a potential inciting role for aeroallergens in patient with EEo, and the subsequent food allergy. BSA is an approximately 67 KDa protein involved both in milk (especially in children) and beef allergy. It explains cross-reactivity among different meats and epitheliums and different mammal´s meat. In our case, the patient´s serum recognized a protein with a molecular weight of 17 kDa, that could correspond to myoglobin, a heat-resistant protein that explains why some patients do not tolerate undercooked or even cooked meat. Thank our study, the patient could discontinued swallowed corticosteroids and avoidance diet, but meats.

## Disclosure of interest

None declared.

